# Evaluation of Colostrum Components and Milking Status Affecting Colostrum IgG Concentration

**DOI:** 10.3390/ani15050718

**Published:** 2025-03-03

**Authors:** Shuji Kayasaki, Hitomi Satoh, Keitaro Oguchi, Kyoko Chisato, Rika Fukumori, Shin Oikawa

**Affiliations:** 1 Nijibetsu Livestock Clinic, Kushiro Central Branch, East Regional Center, Hokkaido Agricultural Mutual Aid Association, 103-1, Line 67, Nijibetsu Genya, Shibecha-cho 088-2461, Hokkaido, Japan; s22041006@g.rakuno.ac.jp; 2 Department of Veterinary Medicine, School of Veterinary Medicine, Rakuno Gakuen University, 582 Bunkyodai-Midorimachi, Ebetsu-shi 069-8501, Hokkaido, Japan; s22241004@stu.rakuno.ac.jp (H.S.); s21761048@g.rakuno.ac.jp (K.O.); k-chisato@rakuno.ac.jp (K.C.); fukumori@rakuno.ac.jp (R.F.)

**Keywords:** cow, colostrum, immunoglobulin G (IgG), bacteria, Brix, milking status

## Abstract

This study investigated IgG concentrations, bacterial contamination, and nutrient components of colostrum on dairy farms in eastern Hokkaido, Japan, to examine the factors making it possible to obtain high-quality colostrum. Colostrum samples were collected from 266 Holstein cows. The general criteria for high-quality colostrum are an IgG concentration of 50 g/L or higher and a total plate count of less than 100,000 CFU/mL. The percentage of samples with the criteria for IgG concentration was low, at 48.9%, but the percentage with the criteria for total plate count was high, at 86.5%. The colostrum Brix value was significantly correlated with the colostrum IgG concentration (r^2^ = 0.233, *p* < 0.001) and provided a rough estimate of that concentration. There was no association between the colostrum IgG concentration and milk components other than protein. For colostrum milking conditions, the time from calving to milking tended to be shorter for high-IgG colostrum than for low-IgG colostrum (*p* < 0.01). The likelihood of high-IgG colostrum increased 1.28-fold with first parity higher and decreased 0.92-fold with a 1 L higher milking volume (*p* < 0.01). The results suggest that, in addition to estimating the colostrum IgG concentration via the Brix value, the likelihood of obtaining high-IgG colostrum is increased by taking into account parity, milking volume, and time from calving to milking.

## Simple Summary

The objective of this study was to examine the factors that contribute to high-quality colostrum in dairy farms in eastern Hokkaido, Japan. The percentage of samples with a high colostrum IgG concentration, an indicator of passive transfer (≥50 g/L), was low, at 48.9%, but the percentage of those with a low total plate count, an indicator of bacterial contamination (<100,000 CFU/mL), was high, at 86.5%. Measurement of colostrum Brix value, a measure of solid component concentration, provided a practical estimate of colostrum IgG concentration. The criteria for obtaining colostrum with high IgG concentration were high parity, low milking volume, and a short time from calving to milking. The estimation of colostrum IgG concentration by Brix meter and the approaching of ideal milking status were thought to lead to the obtaining of high-IgG colostrum.

## 1. Introduction

In calf management, the health implications of colostrum feeding are highlighted. Due to their anatomical structure, newborn calves have no transfer of antibodies via the placenta, and their immune function is underdeveloped, leaving them vulnerable to various diseases and inflammatory conditions. Therefore, postnatal immune acquisition requires the absorption of immunoglobulin (Ig) in colostrum, which helps protect against exposure to common pathogens until 2–4 weeks after birth, when the calf’s immune system is somewhat functional [1]. This is termed passive transfer, and when this transfer is incomplete, the condition is termed failure of passive transfer (FPT). FPT is not a disease, but a condition that predisposes the neonate to the development of disease [2]. Indeed, calves with FPT have been reported to have an increased risk of death and morbidity [3,4,5,6,7,8,9]. In addition to its role as a medium for Ig, colostrum is also a source of nutrition for newborn calves [10]. Among the Ig fractions, IgG is the most abundant antibody in colostrum, accounting for 85–90% of the total Ig [11]. Therefore, colostrum quality is primarily evaluated by IgG, and feeding high-quality colostrum with an IgG concentration above 50 g/L is a factor in the successful transfer of IgG to calves [11,12]. In addition to this, bacterial contamination of colostrum inhibits IgG transport to intestinal cells in the intestinal lumina [13,14,15], making it another leading indicator for judging colostrum quality. As criteria, a total bacteria count of less than 100,000 colony forming units (CFU)/mL and a fecal coliform count of less than 10,000 CFU/mL are recommended [12,16]. A colostrum somatic cell count of less than 400,000 cells/mL is also recommended as an indicator of mastitis causing bacterial contamination and low IgG levels [17]. In addition, colostrum is richer in various components such as maternal leukocytes, growth factors, hormones, cytokines, antimicrobial factors, and nutrients [11,18]. In particular, energy from fat and lactose in colostrum is necessary for thermogenesis and body temperature regulation [11] and is important in seasons with low temperatures. As mentioned above, colostrum management is thought to be important for maintaining calf health. However, to our knowledge, there have been no reports in Japan of investigations into the association between colostrum management and components, and IgG.

The objective of this study was to evaluate the colostrum quality on commercial farms in eastern Hokkaido, Japan, using the quality criteria for the IgG concentration and bacterial counts, and to examine the milking status that is important in obtaining high-quality colostrum.

## 2. Materials and Methods

### 2.1. Farms and Animals

Twenty-three dairy farms in Teshikaga, eastern Hokkaido, where more than 80 calvings per year are expected, were targeted, and colostrum samples were collected from Holstein cows that calved between January and April 2022. Farms were requested to choose 3–5 cows each at first parity, second parity, and third parity and above, and 266 colostrum samples were eventually collected. Annual calving on the target farms ranged from 80–430 cows, and the housing system was free stall on 18 farms and tie stall or stanchion on 5 farms. Grass silage was fed on all farms in the two months prior to calving, hay was fed on 4 farms, and formula feed was fed on 22 farms. Information on the parity, date of insemination, and date of vaccination for the sampled cows was obtained from the database of the Livestock Mutual Aid System of the Agricultural Mutual Aid Association. Vaccination was targeted within the three months before calving, and the only vaccine that fell into this category was bovine diarrhea type 5 inactivated vaccine II (Kyoto Biken Laboratories, Inc., Kyoto, Japan). All animals were treated appropriately following the Laboratory Animal Control Guidelines of Rakuno Gakuen University, which essentially conform to the Guide for the Care and Use of Laboratory Animals of the National Institutes of Health in the United States [19].

### 2.2. Colostrum Collection

The milking supervisor was given a detailed explanation of the colostrum collection procedure and checked the vacuum-pump bucket milker to be used for collection. The colostrum in this study was the first milked after calving. It was confirmed that the collected colostrum was used for the first and second feedings after birth for passive transfer. It was milked from three or more udders, it was limited to individuals with known “date and time of calving” and date of dry-off, and it was measured in containers calibrated to the nearest liter. Colostrum was milked into a bucket milker after pre-milking and teat cleaning. The milking volume and time from calving to milking were not specified, and the farm’s usual methods were used. Colostrum in buckets was first collected in 50 mL sterile containers (Centrifuge Tubes, WATSON, Tokyo, Japan) and promptly stored in the farm refrigerator. The containers were accompanied by a form to be filled with the individual identification number, date and time of calving, date and time of milking, date of dry-off, and milking volume. The colostrum was then collected within 48 h of storage, and the Brix value was measured with a digital colostrum refractometer (PAL-Colostrum, Atago Co., Ltd., Tokyo, Japan) at the time of dispensing into containers for the purpose of measurement. The colostrum for measurement of the somatic cell count was stored at 4 °C and transported to Rakuno Gakuen University once a week. On arrival, the somatic cell count was immediately measured. Colostrum for determining other components and bacterial counts was stored at −20 °C until measurement.

### 2.3. Sample Analysis

#### 2.3.1. Colostrum IgG Concentration

After first seeding polystyrene microwell strips with sheep anti-bovine IgG (1 mg/mL) (Bethyl Laboratories, Inc., Montgomery, TX, USA) diluted 100-fold in IgG buffer (50 mM Tris, 0.14 M NaCl, 0.05% Tween 20, pH 8.0), the strips were coated by reaction at room temperature for 1 h. They were then washed five times by hand and blocked with blocking solution (50 mM Tris, 0.14 M NaCl, 0.05% Tween 20, pH 8.0) for 30 min. Colostrum was thawed at room temperature and immediately placed on ice after thawing and manipulated. Colostrum was diluted 500,000-fold in ELISA coating buffer (carbonic acid-bicarbonate buffer, pH 9.6), seeded with a standard (Bovine IgG, Bethyl Laboratories, Inc.), and reacted for 1 h, and then washed five times by hand. Sheep anti-bovine IgG HRP conjugated (Bethyl Laboratories, Inc.) diluted 100,000 to 150,000-fold was applied and allowed to react at room temperature for 1 h, then washed five times by hand. TMB (Sera Care Life Sciences, Inc., Milford, MA, USA) was applied and allowed to react for 15 min in the dark, and then the absorbance at 450 nm was measured using a microplate reader (iMark, Bio-Rad Laboratories, Inc., Hercules, CA, USA).

#### 2.3.2. Colostrum Components Other than IgG

Colostrum fat, protein, lactose, and solids-not-fat concentrations were determined using a near-infrared spectroscopy analyzer (DA7250, Perten Instruments, Inc., Stockholm, Sweden). Frozen samples were thawed at 42 °C according to the method of thawing frozen samples recommended by QSE GmbH (Wolnzach, Germany). The colostrum somatic cell count was determined by diluting colostrum 20-fold with saline and further diluting it 2-fold with ADAM reagent (SCC-Solution, NanoEntek, Inc., Seoul, Republic of Korea) and measured with an automated somatic cell counter (ADAM-SCC, NanoEntek, Inc.).

#### 2.3.3. Colostrum Bacterial Counts

Colostrum was thawed at room temperature and immediately placed on ice after thawing and manipulated. The total plate count, total coliform count, and *Staphylococcus aureus* count in colostrum were calculated using the plate count method. For the total plate count, colostrum was diluted 10- or 100-fold in saline and seeded onto a Petrifilm RAC Plate (3M Company, Maplewood, MN, USA) at 1 mL and incubated in an incubator (CI−410, Advantech Japan Co. Ltd., Tokyo, Japan) at 35 °C for 24 h. Colony counts were then performed. In the same way, a Petrifilm REC Plate (3M Company) was used for the total coliform count, and a Petrifilm STX Plate (3M Company) was used for the *Staphylococcus aureus* count. When colony counts were low, colostrum stock was applied directly.

### 2.4. Colostrum Quality Criteria

Colostrum IgG concentrations of ≥50 g/L, colostrum bacterial counts of <100,000 CFU/mL for the total bacteria count (this study: total plate count), <10,000 CFU/mL for the fecal coliform count (total coliform count), and <5000 CFU/mL for the other bacterial count (*Staphylococcus aureus* count) are recommended as general colostrum-quality criteria [12,16]. This study was based on these criteria to determine colostrum quality. Colostrum IgG concentrations of ≥50 g/L were classified as high and those of <50 g/L as low, and these were used for analysis.

### 2.5. Statistical Analysis

This study used Excel Statistics ver. 4.07 (SSRI, Tokyo, Japan), a dedicated program for statistical analysis. The statistical significance level was set at less than 5%. The Pearson product–moment correlation coefficient was used to test the correlation between the colostrum IgG concentration and colostrum Brix value, and regression equations were calculated. The normality of the data was confirmed by the Shapiro–Wilk test. Comparisons between high- and low-IgG colostrum were made using the Mann–Whitney U test for continuous variables and the chi-square test for categorical variables. Multivariable analysis with a binomial logistic regression model was used to evaluate the associations of the IgG levels in colostrum with milking status. Dry length was excluded from the explanatory variables as it did not include first parity. The *p* values for the bivariable analyses were provided only as indicators of the strength of association, and no adjustments were made for multiple comparisons. Herd was included as a random effect, and risk factors with *p* < 0.2 for the bivariable analysis were entered into the model and tested using a stepwise method.

## 3. Results

### 3.1. Descriptive Statistics on Colostrum Quality and Milking Status

Descriptive statistics on colostrum quality and milking status are presented in Table 1. In the colostrum components, the median IgG concentration was 49.2 g/L (interquartile range 32.9–71.5 g/L), with ≥50 g/L in 48.9% of the cases. The Brix value was 23.5% (20.5–26.7%). For bacterial counts in the colostrum, the total plate count was 1415 CFU/mL (490–10,500 CFU/mL), with <100,000 CFU/mL in 86.5% of the cases. The total coliform count was 24 CFU/mL (3–429 CFU/mL), with <10,000 CFU/mL in 91.4% of the cases. The *Staphylococcus aureus* count was 310 CFU/mL (110–758 CFU/mL), with <5000 CFU/mL in 95.1% of the cases. With respect to the colostrum milking status, parity was 2.0 (1.0–3.0), milking volume was 5.0 L (3.0–7.0 L), and time from calving to milking was 4.5 h (2.5–8.8 h).

### 3.2. Correlation Between Colostrum IgG Concentration and Colostrum Brix Value

**Figure 1:**
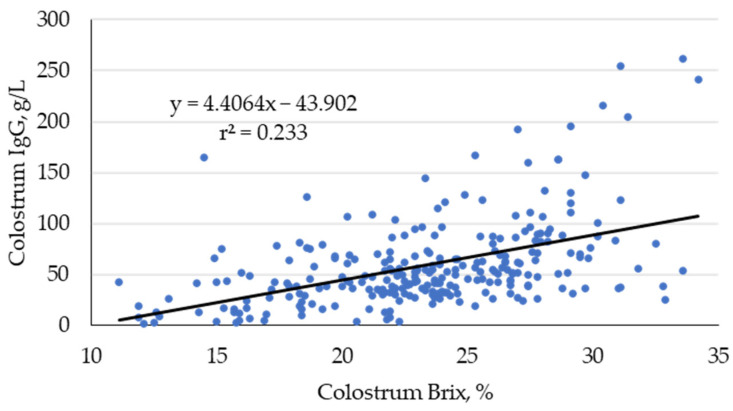
Scatter plots and the approximate curves of the colostrum IgG concentration (g/L) and colostrum Brix value (%) in 266 samples.

Scatter plots and the approximate curves of the colostrum IgG concentration (g/L) and colostrum Brix value (%) are shown in Figure 1. The regression equation of the single regression analysis with the IgG concentration as an objective variable and Brix value as an explanatory variable was as follows: IgG (g/L) = 4.4064 × Brix (%) − 43.902 (r^2^ = 0.233, *p* < 0.001). From this equation, the colostrum Brix value corresponding to the colostrum IgG concentration of 50 g/L was calculated to be 22.8%.

### 3.3. Comparison of Colostrum Quality Between High- and Low-IgG Colostrum

A comparison of colostrum quality between high- and low-IgG colostrum is shown in Table 2. In colostrum, the Brix value, protein, and solids-not-fat percentage were significantly higher (*p* < 0.001) for high-IgG colostrum than for low-IgG colostrum. There were no differences between the two groups in other items.

### 3.4. Association of Colostrum IgG Concentration with Milking Status (Binomial Logistic Regression Model)

A comparison of milking status between high- and low-IgG colostrum is shown in Table 3. Parity was significantly higher for high-IgG colostrum than for low-IgG colostrum (*p* < 0.05). On the other hand, milking volume was significantly lower for high-IgG colostrum (*p* < 0.05), and the time from calving to milking was significantly shorter (*p* < 0.01). A binomial logistic regression model for the association of the colostrum IgG concentration with milking status is shown in Table 4. The regression equation for the binomial logistic regression analysis was finally populated with parity, milking volume, and time from calving to milking. Higher parity significantly increased the likelihood of high-IgG colostrum (odds ratio 1.28, *p* < 0.01). Higher milking volume (L) significantly decreased the likelihood of high-IgG colostrum (odds ratio 0.92, *p* < 0.05). The likelihood ratio of the regression equation was significant (*p* = 0.0016).

## 4. Discussion

The percentage of colostrum IgG concentrations above 50 g/L in this study was 48.9%, which was in the range of previous reports on dairy cows (42.2–90.4%) [20,21,22,23,24], but tended to be lower, indicating room for improvement in the colostrum IgG level. On the other hand, the percentage of total plate counts of less than 100,000 CFU/mL was 86.5%, higher than in previous reports on dairy cows (using frozen samples: 19–65.6%) [20,23,25,26,27,28,29] and the percentages of total coliform counts of less than 10,000 CFU/mL and *Staphylococcus aureus* counts of less than 5000 CFU/mL were similarly high. It has been reported that colostrum stored in refrigeration has a lower increase in bacterial counts than colostrum stored at room temperature [28,30] and that colostrum collected directly from the mammary gland has low bacterial counts, but bacterial contamination occurs during the milking process [26,30]. In this study, the samples were stored in a refrigerator after collection and were collected mainly during the winter, so the effect of temperature was assumed to be small. On the other hand, the total plate count was lower than the criteria in many of the samples despite being taken from milking buckets, indicating that proper disinfection of equipment was excellent in the milking hygiene management on the farm.

There are reports both that colostrum Brix values of dairy cows provide reliable estimates of IgG concentrations [24,31,32] and that colostrum Brix values do not correlate with IgG concentrations [33]. The colostrum IgG concentration in this study was significantly correlated with the Brix value (r^2^ = 0.23, *p* < 0.001). The colostrum IgG concentration was estimated to be 50 g/L when the Brix value was 22.8%, which is comparable to previous reports of Brix criteria (18–22%) [24,31,32]. On the other hand, the correlation between Brix values and IgG concentrations (R^2^ = 0.233) tended to be lower than that reported in other studies (R^2^ = 0.397–0.960) [24,31,32]. This may be due, in part, to the difference in measurement methods between this study, which used an enzyme-linked immunosorbent assay, and other studies that used radial immunodiffusion or turbidimetric immunoassays for IgG measurement. However, the results suggest that the estimation of IgG concentrations by Brix values was shown to have a certain usefulness. Additional factors, such as milking conditions, need to be taken into account to collect colostrum IgG concentrations of ≥50 g/L.

Regarding colostrum components, the protein and solids-not-fat percentages tended to be higher for high-IgG colostrum than for low-IgG colostrum; this was attributed to the amount of IgG. No association between the IgG concentration and other nutrients was observed. These results indicated that high-IgG colostrum did not guarantee enrichment with fat and lactose. In addition, the fat concentration, which is an important source of energy, was shown to be highly variable by the interquartile range.

Among the colostrum milking status criteria, parity tended to be higher for high-IgG colostrum than for low-IgG colostrum. These results support the report that the colostrum IgG concentration in dairy cows increases with parity and tends to be lower in first-parity cows [21,22,23,24,34,35,36,37,38,39,40]. This may be due to the fact that older cows were exposed to farm-specific pathogens for greater periods of time [11].

There are some reports that colostrum IgG concentrations in dairy cows decrease with increases in the weight and volume of collected colostrum [24,35,38,41,42], while other reports assert that there is no association [33,43,44]. In this study, lower milking volume tended to be associated with high-IgG colostrum, suggesting that lower milking volume is more effective in facilitating the collection of high-IgG colostrum. Godden and Hazel [45] reported different IgG concentrations depending on milking fractions from cisternal to alveolar with regard to colostrum collection. Since this study did not stipulate whether or not the samples were completely milked when collected, it was inferred that both cases in which colostrum was completely milked, and those in which less colostrum was intentionally milked, were included. Therefore, it is considered necessary to investigate the effect of adjusting the milking volume of colostrum on the IgG concentration in the future.

It has been reported that colostrum IgG concentrations in dairy cows decrease with time from calving to milking [21,24,36,42]. In this study, the time from calving to milking tended to be shorter for high-IgG colostrum than for low-IgG colostrum, suggesting that milking as early as possible after calving is more effective in collecting high-IgG colostrum.

Logistic regression analysis identified parity and milking volume as important factors affecting the colostrum IgG concentration. The likelihood of high-IgG colostrum increased 1.28-fold with first parity higher and decreased 0.92-fold with a 1 L higher milking volume. Overall, the results suggest that in addition to the estimation of colostrum IgG concentration by Brix value measurement, the likelihood of obtaining high-IgG colostrum is increased by taking into account parity, milking volume, and time from calving to milking. The results also indicated that high-IgG colostrum did not guarantee an increase in milk components other than protein.

## 5. Conclusions

This study revealed the actual condition of colostrum quality on dairy farms in eastern Hokkaido, Japan. Bacterial contamination of colostrum was low and hygienic, but IgG concentrations were inadequate. Some usefulness in the estimation of IgG by Brix values was confirmed. In order to obtain high-IgG colostrum, it was important to take into account the collection factors of parity, milking volume, and time from calving to milking. Although this study focused on the short-term perspective of milking status, it highlighted the need to consider long-term measures for obtaining high-IgG colostrum, such as prepartum management and genetic improvement.

## Figures and Tables

**Table 1:** Descriptive statistics on colostrum quality and milking status in 266 samples.

Item	Median	Minimum	Interquartile Range	Maximum	Mean ± Standard Deviation
Components					
IgG, g/L	49.2	1.9	32.9–71.5	261.8	58.7 ± 42.5
Brix, %	23.5	11.1	20.5–26.7	34.2	23.3 ± 4.7
Protein, %	13.5	3.9	11.2–16.9	29.8	13.8 ± 4.3
Lactose, %	2.7	1.3	2.3–3.0	4.0	2.7 ± 0.5
Fat, %	5.9	1.0	4.2–8.0	18.6	6.2 ± 2.9
Solids-not-fat, %	18.0	9.9	15.7–21.4	38.8	18.5 ± 4.4
Somatic cell count, 10^3^ cells/mL	1380	60	680–3205	45,500	3234 ± 5272
Bacterial counts					
Total plate count, CFU/mL	1415	2	490–10,500	295,000,000	1224,275 ± 18,089,636
Total coliform count, CFU/mL	24	1	3–429	370,001	7733 ± 38,358
*S. aureus* count, CFU/mL	310	0	110–758	198,000	2275 ± 13,189
Milking status					
Parity	2.0	1.0	1.0–3.0	8.0	2.3 ± 1.4
Milking volume, L	5.0	0.5	3.0–7.0	25.0	5.8 ± 3.9
Time from calving to milking, h	4.5	0.0	2.5–8.8	30.0	6.3 ± 5.3
Gestation length, days	279	268	276–282	311	280 ± 5
Dry length, days ^a^	59	21	51–63	171	60 ± 18

^a^ Multiparous cows (n = 178).

**Table 2:** Comparison of colostrum quality between high- and low-IgG colostrum in 266 samples.

Item	Colostrum IgG Concentration ^a^				*p*-Value
	High		Low		
	Median	Interquartile Range	Median	Interquartile Range	
Components					
Brix, %	25.6	22.9–27.9	22.1	18.4–24.0	<0.001
Protein, %	15.5	12.6–18.2	12.1	9.5–14.8	<0.001
Lactose, %	2.7	2.3–3.0	2.7	2.4–3.0	0.144
Fat, %	5.7	3.8–7.8	6.2	4.5–8.0	0.131
Solids not fat, %	20.0	16.9–23.4	16.5	14.2–19.4	<0.001
Somatic cell count, 10^3^ cells/mL	1510	760–3590	1300	635–2705	0.233
Bacterial counts					
Total plate count, CFU/mL	1130	470–6825	1785	490–23,525	0.119
Total coliform count, CFU/mL	25	3–256	22	2–599	0.655
*S. aureus* count, CFU/mL	285	110–620	330	106–938	0.314

^a^ High (n = 130): ≥ 50 g/L; Low (n = 136): <50 g/L; The Mann–Whitney U test was performed between high- and low-IgG colostrum.

**Table 3:** Comparison of milking status between high- and low-IgG colostrum in 266 samples.

Variable	Colostrum IgG concentration ^a^						*p*-Value
	High			Low			
	Median	Interquartile Range	(Mean ± Standard Deviation) ^f^	Median	Interquartile Range	(Mean ± Standard Deviation) ^f^	
Milking status							
Parity ^d^	2.0	1.0–3.0	2.5 ± 1.4	2.0	1.0–3.0	2.1 ± 1.3	0.022
Milking volume, L ^d^	5.0	3.0–6.0	5.2 ± 3.4	5.0	3.0–8.0	6.3 ± 4.2	0.024
Time from calving to milking, h ^d^	4.0	2.0–7.9	5.7 ± 5.3	5.8	3.0–10.0	6.9 ± 5.3	0.008
Gestation length, day ^d^	279	276–283	279 ± 5	280	277–282	280 ± 6	0.349
Dry length, day ^b, d^	59	51–63	58 ± 12	59	53–63	61 ± 22	0.807
Vaccination ^c, e^							
Yes	14			19			0.429
No	116			117			

^a^ High (n = 130): ≥50 g/L; Low (n = 136): <50 g/L; ^b^ Multiparous cows (n = 178); ^c^ Bovine diarrhea type 5 inactivated vaccine II (Kyoto Biken Laboratories, Inc.). Vaccination within 3 months before calving; ^d^ The Mann–Whitney U test was performed between high- and low-IgG colostrum; ^e^ Values are number of samples. The chi-square test was performed between high- and low-IgG colostrum; ^f^ (Mean ± standard deviation) is for reference.

**Table 4:** Binominal logistic regression model for the association of the colostrum IgG concentration with milking status.

Variable	Estimate	Robust Standard Error	Odds Ratio	95% Confidence Interval	*p*-Value
Intercept	0.09	0.33	1.10	0.58–2.09	0.774
Milking status					
Parity	0.25	0.10	1.28	1.06–1.55	0.009
Milking volume, L	−0.08	0.04	0.92	0.86–0.99	0.024
Time from calving to milking, h	−0.04	0.02	0.96	0.91–1.01	0.091

In this model, “high-IgG concentration (≥50 g/L) or not” was used as a response variable and milking status as the explanatory variable.

## Data Availability

The data presented in this study are available upon request from the corresponding author.
